# The Innovative Medicines Initiative: a Public Private Partnership Model to Foster Drug Discovery

**DOI:** 10.5936/csbj.201303017

**Published:** 2013-11-27

**Authors:** Elisabetta Vaudano

**Affiliations:** a Innovative Medicines Initiative, Avenue de la Toison d'Or 56-60, B-1060, Brussels, Belgium

**Keywords:** drug efficacy, drug safety, knowledge management, pharmaceutical industry, bottlenecks, attrition rate

## Abstract

The Innovative Medicines Initiative (IMI) is a large-scale public–private partnership between the European Commission and the European Federation of Pharmaceutical Industries and Associations (EFPIA). IMI aims to boost the development of new medicines across Europe by implementing new collaborative endeavours between large pharmaceutical companies and other key actors in the health-care ecosystem, i.e., academic institutions, small and medium enterprises, patients, and regulatory authorities. Currently there are more than 40 IMI projects covering the whole value chain of pharmaceutical R&D, but with a strong focus on drug discovery, as an ideal arena where the PPP concept of pre-competitive collaboration can rapidly deliver results. This article review recent achievements of the IMI consortia of relevance to drug discovery, providing proof-of-concept evidence for the efficiency of this new model of collaboration.

## Introduction

Investment in pharmaceutical research and development (R&D) has increased substantially in the last decades. Disappointingly, this has not translated into a corresponding increase in output in terms of new drugs approved, indicating that therapeutic innovation has become more challenging [1]. At the same time healthcare costs are steadily spiralling, fuelled by the ageing of the population and the parallel rise in the chronic disease burden. European healthcare systems are therefore faced with the challenge of delivering optimal treatment, with both improved outcomes and reductions in costs, to needy patient populations [2, 3].

Late stage drug development is the most costly area of drug R&D and also shows the highest attrition rate [4], with severe financial implications. Achieving a decrease in late-stage drug failure will require the optimisation of the drug discovery phase to increase both the probability of success and the number of New Chemical Entities (NCE) in pharmaceutical pipelines [5]. Drug discovery programmes are initiated and driven by the belief that an efficacious new drug can be identified and made available to suffering human patients. Nevertheless, even if a drug lead is identified, the path to clinical drug candidate is the most idiosyncratic segment of drug discovery and development, with frequent setbacks and failures which defy generalisation [6].

## Novel collaborative models are needed for pharmaceutical R&D

Many pharmaceutical companies are realising that a paradigm shift in the industry's research and development (R&D) strategy is the only way of reversing the currently ongoing negative trend. Novel strategies based on an integrated and collaborative approach are required, building on innovation and leveraging on the strengths and input of all stakeholders in the health system with the shared goal of delivering effective and sustainable healthcare solutions for society [7, 8].

One approach that has gained momentum in recent years is the establishment of precompetitive public–private partnerships (PPPs), as ideal vehicles to solve issues that are too large for single organisations to effectively address alone. Such models rely on open innovation networks that link up the know-how and resources of the pharmaceutical industry with external pools of knowledge, especially in universities and biotechnology companies [9, 10].

This concept has been pioneered in the area of neglected diseases (e.g. see http://www.mmv.org/about-us) where public private partnerships have been vital to fill the development pipeline [11]. In the last years several PPPs in the pharmaceutical sector have been launched [12, 13]. In particular, in the USA PPPs have been fostered by the US Food and Drug Administration, under the umbrella of the Critical Path Initiative [14], and by the National Institutes of Health (NIH) [9, 15]. In Europe, the Innovative Medicines Initiative (IMI), a public-private partnership between the EU and the European Federation of Pharmaceutical Industries and Associations, is a prototypic example of an organisation created to support open innovation and pre-competitive research in the pharmaceutical sector [16]. IMI-sponsored activities are conducted by consortia that bring together pharmaceutical companies, small- and medium-sized enterprises (SMEs) and partners from the public sector. EFPIA-affiliated pharmaceutical companies invest in the form of ‘in kind’ contributions by committing resources, providing access to data sets and infrastructures, and sometimes through direct monetary contributions. At least two companies actively participate in each project. This industry investment is matched by funds from the European Commission's Seventh Framework Programme for Research and Technological Development (FP7) to other consortium members, including academic teams, SMEs, patients’ organisations, regulatory agencies and other non-profit institutions.

## IMI contribution to ease the bottlenecks of drug discovery

The Innovative Medicines Initiative is promoting partnerships to tackle bottlenecks in drug discovery and development and confront areas of healthcare need that are of high priority to society, leading to the more efficient discovery and development of better and safer medicines for patients. Currently there are more than 40 IMI projects covering the whole value chain of pharmaceutical R&D, but with a strong focus on drug discovery, as an ideal arena where the PPP concept of pre-competitive collaboration can rapidly deliver results (for a full list of IMI projects please visit http://www.imi.europa.eu/content/ongoing-projects).

The starting point of drug discovery is the decision on the strategy to take for disease modulation. Since the dawn of the genomics era in the 1990s, the main focus of drug discovery has been on drug targets, which are typically proteins that appear to have a key role in disease pathogenesis. Thus target discovery represents typically the first milestone in drug discovery. The recent advances in the understanding of the molecular basis of diseases and the completion of the Human Genome Project have dramatically expanded the number of plausible therapeutic targets for the development of innovative drugs. This progress has enabled the development of drugs that have revolutionised e.g. cancer treatment (e.g. imatinib, [17]. However targets are frequently found to have poor drugability, and the current number of novel validated molecular drug targets associated with disease pathology is still very limited, making the probability of identifying suitable pharmacological modulators stubbornly low.

A key challenge for drug developers is therefore, on the one hand, how to most efficiently and successfully choose the best targets from the plethora of candidates generated by several large ‘omics’ efforts and further develop them into innovative, safe and efficacious drugs. The problem is especially severe in complex diseases such for example diabetes and its complications, the disorders of the nervous system, and cancer.

On the other hand, for many diseases it may be beneficial to target broader pathways or phenotypes instead of single targets. Indeed a recent analysis revealed that phenotypic screening has been more successful than target-driven screening in delivering first-in-class medicines [18], although also this approach has several pitfalls. Ideally, an appropriate combination of both approaches should be found, to enable researchers to move forward with the best possible candidates [18, 19], and create an effective R&D productivity strategy delivering innovative products with high-quality information [5].

In this context the big future wins both for industry and for the patients lie more likely in the identification of genes and protein molecules associated with disease subtypes and to specific pathophysiological events and pathways (molecular phenotyping), or related to common causes of drug attrition problems (e.g. cardiotoxicity), and in the their successful translation to reliable predictions of future events and better estimate of benefit and risk in clinical studies. This requires, among other things, significant progress on methods and tools for defining molecular mechanisms of action, and for better translation of results from research to the clinic [12, 20]. It is unlikely that any one company working in isolation can achieve this goal. A further key issue, slowing down progress, is the limited robustness and reproducibility of published academic preclinical studies which should pave the way to the development of new drugs [21–24]. Clearly, a concerted action of all stakeholders, public and private, is needed to insure success in such complex endeavour.

IMI partnerships are successfully contributing to easing these challenges. In this review some examples are provided of outputs of IMI projects fostering drug discovery in major disease areas with a high impact on the health care burden, as well as developing knowledge management tools for prediction of drug safety and efficacy. Finally, some details are provided on two most recent initiatives with broad potential impact on early drug discovery.

## Drug discovery for the treatment of micro- and macrovascular complications of diabetes

Diabetes mellitus is a lifelong, incapacitating disease affecting multiple organs. The disease is associated with devastating chronic complications including coronary heart disease, stroke and peripheral vascular disease (macrovascular disease) as well as microvascular disorders leading to damage of kidneys (nephropathy) and eyes (retinopathy). After 10 years of diabetes, over 70% of diabetic patients have some degree of complications [25]. These complications impose an immense burden on the quality of life of the patients. In particular diabetic nephropathy (DN) is a complication that develops in approximately 25–40% of diabetic patients and is the leading cause of end-stage renal disease (ESRD) in the developed world. Currently, 44% of the new cases of ESRD in the US annually are attributable to DN [26]. A better understanding of the causal factors of DN and its pathogenesis may lead to new strategies to decrease its incidence, pre-emptively treat the disorder, and attenuate morbidity and mortality, and as such would be a valuable contribution to global public health. One of the objectives of the IMI SUMMIT project (http://www.imi-summit.eu) is to identify novel genetic markers predicting susceptibility to chronic diabetic complications to provide insights into potential targets for biomarker and therapeutics development. To this end the SUMMIT consortium has assembled and phenotyped the largest genome-wide association study (GWAS) database for type 1 diabetes patients with diabetic nephropathy (DN). By mining this unique resource of carefully characterised patients, the consortium found that several previously reported genetic associations with DN in type 1 diabetes could not be replicated in a large, homogeneous sample of subjects [27]. In addition SUMMIT identified new susceptibility loci associated with kidney disease in type 1 diabetes, which represent new signals in the pathogenesis of DN [28]. The results of the consortium's work underscore the need to apply stringent statistical thresholds of significance, maximise power through meta-analysis of all available data, and seek replication in independent samples to obtain data solid enough for supporting target drug discovery, as previously indicated by other authors [29] and ideally fostered via the PPP collaborative model. Other parts of the SUMMIT project are gathering lipidomics, metabolomics and other biomarker data which will be linked to phenotypic data of the vascular complications of diabetes. Taking advantage of the computational tools developed by the consortium [30] and novel translatable animal models [31] SUMMIT will develop a network model to replace a single target view with a system-wide perspective of diabetes complications to support new strategies for drug developers in the area.

## Drug discovery for chronic pain

One in five adults suffers from chronic pain. This constitutes a major cause of long-term sick leave and forced early retirement, placing a great financial burden on both individuals and healthcare systems. Despite extensive research programmes by biopharmaceutical companies and academia, there remains a need for treatments that are more effective and have fewer side-effects [32]. The discovery of new pain mediators amenable to manipulation is likely to yield much needed novel therapeutic targets. Approaches to finding pain mediators encompass high throughput genetic (GWAS) and expression profiling (transcriptomics, proteomics and lipidomics) of chronic pain patients and experimental models. A key challenge is that pain mechanisms are not always controlled in the same ways in preclinical species and in humans [33]. This has often prevented the efficient translation of results from the preclinical field to the clinic. The EUROPAIN consortium (http://www.imieuropain.org/) has established an international team of leading researchers and clinicians from both academia and industry to undertake multidisciplinary translational research for chronic pain. The consortium is leading a major effort for the standardized phenotyping of pain symptoms and sensory signs in patients and human surrogate models [34], and development of translational animal models and validated outcome measures of evoked and spontaneous pain behaviour that more accurately depict similar outcome measures used in humans, including affective components of pain [35]. These models are rigorously standardised via interaction with the industry partners of the consortium. By using computational approaches to integrate datasets including sensory phenotype as well as the outputs of high throughput technologies in humans and rodents, EUROPAIN is making progress on deciphering the neurobiology of pain, something which has already lead to the identification of an attractive potential drug target [36, 37].

## Drug discovery for Autism Spectrum Disorder

Autism spectrum disorder (ASD) is a common neuro-developmental disorder affecting approximately 1% of children. Autism has life-long consequences with a range of impacts on the health, economic well-being, social integration and quality of life of individuals with the disorder, and also on their families and potentially the rest of society [38](Knapp et al, 2009). A recent report [39] estimates that the annual cost to governments for care of ASD individuals will increase by 2028 to an estimated $18 billion in the US alone.

Discovering novel treatments for ASD is a challenge. Its aetiology and pathology remain largely unknown, the condition shows wide clinical diversity, and case identification is still based solely on symptomatology. However there has been recent significant progress in the understanding of the biology underpinning ASD. This creates hope that harnessing these new developments can lead to novel treatments driven by the likely biological basis of this condition [40, 41].

The IMI EU-AIMS project (http://www.eu-aims.eu/#) involves a novel collaboration between organisations representing affected individuals and their families (Autism Speaks), academia and industry that for the first time in the world are joining forces to develop the infrastructure underpinning new treatments for autism and to develop and assess novel treatment approaches for the condition [42].

Although this is a very young initiative, which only started working in April 2012, the pre-competitive collaborative research approach of the project is already bearing fruit.

Twin and family studies have demonstrated that heritability plays a large role in autism. Several risk gene variants have now been identified that significantly contribute to ASD susceptibility, many of which are linked to synaptic functioning, excitation--inhibition balance, and brain connectivity [43, 44]. EU-AIMS partners are whole genome sequencing a large number of individuals with autism and their parents. The results of this effort will provide an overview of the de novo and segregating mutations contributing to the risk of autism. The first results of these efforts have already demonstrated a role for father's age in such risk [45].

Multiple genetic syndromes include ASD symptoms as part of a broader pattern of dysmorphology and medical morbidity [46]. Studies of these syndromic forms of ASD have provided significant insight in its underlying biology. For example in fragile X syndrome, about 25% of patients meet diagnostic criteria of autism [47]. In fragile X, the key defect in synaptic transmission is elevated group I metabotropic glutamate receptor-dependent synaptic plasticity (mGluR-LTD). EU-AIMS partners have discovered an unexpected convergence of synaptic pathophysiology in a non-syndromic form of autism with that in fragile X syndrome. Furthermore they have demonstrated in a novel knock–out mouse model that the phenotype could be rescued, highlighting the possibility of reverting neuronal circuit alterations in autism after completion of development [48]. The PPP nature of the consortium will now allow the rapid incorporation of these new finding into the drug discovery strategy of the companies involved, taking advantage as well of the novel translational animal models developed by the consortium.

## Drug Discovery for cancer

There is a huge cancer burden in Europe. In 2006 there were an estimated 3,191,600 new cases of cancer diagnosed, and 1,703,000 deaths from the disease [49]. While it is widely recognised that major advances have been made both in the understanding of the disease and also in the treatment of many forms of cancer, a large number of anticancer drugs still fail due to a lack of efficacy in late stage trials. Improvements in understanding of the underlying biology of cancer and the development of new models for target validation is essential for supporting the significant advances required to improve the quality of cancer drug discovery. Historically in cancer R&D, targets have been inappropriately selected or validated due to the use of reductionist models which do not represent the complexity of tumours *in situ*, resulting in failure in the clinical setting [50].

The PREDECT IMI project (http://www.predect.eu/about/) is developing improved *in vitro* and *in vivo* models to support target identification and target validation in cancer with greater capacity to predict outcomes in the human disease. The project approach has recently led to a remarkable breakthrough. It is well established that estrogens and progesterones are major drivers of breast development but also promote carcinogenesis in this organ. ‘Receptor activator of nuclear factor κB ligand’ (RANKL) has been identified as a pivotal paracrine mediator of progesterone function in mouse mammary gland development and mammary carcinogenesis [51, 52]. Whether the factor has the same role in humans is of clinical interest because an inhibitor for RANKL, denosumab, is already used for the treatment of bone disease [53] and might benefit breast cancer patients.

Taking advantage of a novel *ex vivo* model to study hormone action in the human breast the PREDECT consortium has been able to demonstrate that RANKL-mediated hormonal control mechanisms are conserved across species [54]. This makes RANKL a potential target in breast cancer treatment and prevention, with important clinical applications.

## Boosting knowledge management for better and earlier prediction of drug safety and efficacy

The *in silico* prediction of biological phenomena on the basis of structural information of the drug candidates shows attractive results in the case of some molecular pharmacology parameters (e.g., affinities for particular biological targets), in some pharmacokinetics-related properties, or for a few toxicological endpoints such as mutagenicity [55, 56]. Nevertheless, the *in silico* prediction of most of the important *in vivo* outcomes on the basis of information available in early stages of the drug discovery/development is still far from being a reality. Early *in silico* prediction of *in vivo* toxicological outcomes would increase the quality of drug candidates and ensure a lower attrition rate during subsequent phases of the drug development pipeline. This would also reduce the number of animals to be used in *in vivo* toxicological studies.

The IMI eTOX project (http://www.etoxproject.eu/) is developing innovative methodological strategies and novel software tools to better predict the toxicological profiles of new molecular entities in early stages of the drug development pipeline. One of the pillars of the eTOX project is the integrated use of public and private data. With respect to private data, the project is collating information from legacy reports obtained in chronic toxicity studies carried out in the 13 pharmaceutical companies that participate in the project. In the case of public data, eTOX after identification and integration of currently available data in the public domain is working in evaluating the data quality and its coverage regarding toxicity issues, in order to gather and process only data suitable for the building of predictive models. The main result of this effort is a large web-based structured library containing links to articles of toxicological relevance (data that can be used for modeling purposes, computational models, and toxicity mechanisms), public databases, standardised vocabularies and modeling tools. The library has been made public at the eTOX website, where it is updated on a monthly basis, constituting a useful resource for the in silico toxicity prediction of novel drug candidates [57]. The project is also delivering new computational models for early assessment of drug toxicity, suitable for preliminary screening in lead discovery, before a compound is physically available. One of these models is a multiscale simulation system aiming to produce a better cardiotoxicity assessment. The model has been tested in predicting the cardiotoxic effect of several compounds, including some examples in which classic potassium channel hERG (human ether-a-go-go-related gene, K(v)11.1)-based models produce false positive or negative results, yielding correct predictions for all of them [58]. Another model developed by the project has been shown to be useful for the prediction of drug induced nausea and vomiting, a common side effect limiting the therapeutic value of many drugs [59]

Pharmaceutical companies currently expend significant efforts integrating the vast amount of data publicly available into internal architectures. This is particularly the case with pharmacological data. Such problems could be reduced, and R&D efficiency increased, by access to a comprehensive database of pharmacological data to help initial drug screening stages and limit expensive clinical trial failure. The IMI Open PHACTS project (http://www.openphacts.org/) integrates multiple publicly-available databases, creating links between the data present, allowing access to a vast data resource in a stable and rigorous infrastructure. The provenance of all data is easily assessed, and traceable back to the parent database, allowing the data quality to be evaluated [60]. Furthermore the project has developed several applications to query the data for different purposes (PharmaTrek, ChemBioNavigator, GARField, Target Dossier, Utopia Documents)

The Open PHACTS discovery platform has been built to answer critical pharmacological questions as defined by academic and pharmaceutical industry scientists. In addition to reducing barriers to drug discovery within the pharmaceutical industry, the Open PHACTS platform allows scientists in academia and smaller companies unprecedented access to an integrated database of pharmacological information.

## Public Private Partnership approaches for enhancing lead discovery and optimisation

IMI has recently launched two novel and challenging initiatives with the potential of a broad impact on early drug discovery. These are the ‘European Lead Factory’ (ELF) and ‘Kinetics for Drug Discovery’ (K4DD) projects.

High-throughput screening (HTS) of comprehensive collections of chemical compounds has proven to be a major avenue towards the identification of novel candidates for further development into lead structures and drug candidates, i.e. NCEs. Although pharmaceutical companies have built up large libraries of compounds over the years, access to these collections has been tightly restricted to in-house use by the owners. Meanwhile, the academic community is becoming increasingly interested in HTS. Triggered by the NIH roadmap in 2004 in the United States, this area has recently seen active growth also in Europe, e.g. in the EU OPENSCREEN project. Still, public compound collections tend to be rather small and expertise in the area is scattered across many institutions. As a result, few public drug targets have been screened against large, high-quality compound libraries. This has hampered efforts to generate promising leads for the development of innovative drugs. The IMI-funded European Lead Factory (ELF) (http://www.europeanleadfactory.eu/) will provide an industry-like small molecule discovery platform to public investigators [61]. Furthermore, as the University of Oxford is a partner in the ELF, it will leverage the experience and know-how of the Structural Genomics Consortium [62, 63], another important PPP in the field.

ELF builds on a unique, comprehensive, high-quality compound collection contributed by EFPIA participants, i.e. the Pharma Consortium Collection, totalling over 300 000 compounds. To this will be added an estimated additional 200 000 novel compounds generated by public partner contributions during the project, resulting in a unique Joint European Compound Collection with some 500 000 compounds. This unique collection might then contain more three-dimensional molecular structures, as is the case in nature, since to date the chemical processes used by the pharmaceutical industry have tended to yield planar structures. Screening of these previously-safeguarded corporate compound libraries against competitors’ targets and targets from public sources may result in otherwise inaccessible valuable lead structures that could ultimately result in the development of novel treatment options for patients.

There is mounting evidence that the often ignored kinetic aspects of the interaction between a small molecule drug and its protein target in the body are highly relevant for *in vivo* efficacy and clinical success [64–66]. It may even be so that lack of understanding of these kinetic aspects is one of the main reasons for the high attrition rates in drug discovery and development. Therefore, there is a growing need for knowledge of the kinetics of binding, as it may play a crucial role in drug efficacy and safety. Interestingly, recent overviews showed that many approved and successful drugs favour certain kinetic aspects [67]. Today, a lot of the expertise and data on binding kinetics is scattered across numerous smaller projects, institutions and organisations.

By bringing together these diverse groups, the IMI project ‘Kinetics for Drug Discovery’ (K4DD) (http://www.k4dd.eu/) is set to give a major boost to this important area of drug development. The first goal of the K4DD team is to enhance the understanding of binding kinetics. Ultimately, the project aims to develop a range of robust techniques, methods and models that could be easily incorporated into the drug development pathway and enable scientists and drug designers worldwide to reliably predict a molecule's kinetic properties (its ‘kinotype’). This information will allow drug developers to more easily determine the safety and efficacy of a molecule. In the long run, this will weed out ineffective or unsafe molecules earlier in the drug development process.

## Conclusions

Drug discovery key challenges reside in a still incomplete understanding of the human diseases and mechanisms investigated, followed often by the selection of non-optimal drug molecules for further development. Public-private partnerships represent attractive means to leverage resources dispersed across industry, academia, and voluntary health organisations in order to address its multiple challenges, in an era of constrained resources. The reported achievements of IMI projects demonstrate that this approach can lead to significant advances for the development of innovative drugs.
